# Applied Anatomy of the Lungs and Pleural Membranes

**Published:** 1912-09

**Authors:** 


**Applied Anatomy of the Lungs and Pleural Membranes.** By J. STUART DICKEY, M.D., B.Ch. Pp. viii, 127. Belfast: Alexander Mayne & Boyd. 1911.

A knowledge of the applied anatomy of the lungs and pleura is an essential preliminary to the study of physical signs and the physical diagnosis of lung diseases. Unfortunately this is too often forgotten, and students are apt to think that they can enter upon the most difficult problems of physical diagnosis with the vaguest ideas as to the anatomy of the thoracic viscera, and especially of their relations to the chest wall. We might add that older practitioners also sometimes make mistakes in diagnosis from forgetfulness or ignorance of anatomical relations. This little book gives a very good account of the subject, and we think it will be found a valuable addition to the practitioner's bookshelf. It deals fully with the position and anatomical relations of the apices of the lungs, and of the relations of the clavicle to the lungs. The sections on the lymphatics and lymphatic glands of the pleura lungs and chest wall contain much valuable information, which it is of great practical importance to bear in mind. The descriptions of the pleural cavities and of the internal structures corresponding to certain surface landmarks are also very well put. There is a useful section on the routes of infection in pulmonary tuberculosis. Numerous figures of dissections and of sections of the thorax at different levels add to the value of the work. These figures are fifty in number, and are clear and well executed. We recommend this as a decidedly useful book, and an aid to accuracy in physical diagnosis of lung diseases.

